# Selenocysteine, Pyrrolysine, and the Unique Energy Metabolism of Methanogenic Archaea

**DOI:** 10.1155/2010/453642

**Published:** 2010-08-17

**Authors:** Michael Rother, Joseph A. Krzycki

**Affiliations:** ^1^Institut für Molekulare Biowissenschaften, Molekulare Mikrobiologie & Bioenergetik, Johann Wolfgang Goethe-Universität, Max-von-Laue-Str. 9, 60438 Frankfurt am Main, Germany; ^2^Department of Microbiology, The Ohio State University, 376 Biological Sciences Building 484 West 12th Avenue Columbus, OH 43210-1292, USA

## Abstract

Methanogenic archaea are a group of strictly anaerobic microorganisms characterized by their strict dependence on the process of methanogenesis for energy conservation. Among the archaea, they are also the only known group synthesizing proteins containing selenocysteine or pyrrolysine. All but one of the known archaeal pyrrolysine-containing and all but two of the confirmed archaeal selenocysteine-containing protein are involved in methanogenesis. Synthesis of these proteins proceeds through suppression of translational stop codons but otherwise the two systems are fundamentally different. This paper highlights these differences and summarizes the recent developments in selenocysteine- and pyrrolysine-related research on archaea and aims to put this knowledge into the context of their unique energy metabolism.

## 1. Introduction

Expansion of the amino acid repertoire of proteins beyond the 20 “canonical” amino acids is a phenomenon observed almost 50 years ago [1]. Numerous modifications of the carboxyl- or amino-terminals or the individual side chains of amino acids after ribosomal synthesis of the respective polypeptide had finished were identified and the biosynthetic path elucidated (reviewed in [2]). It is thus not surprising that a similar process was assumed when selenocysteine, 2-selenoalanine, was discovered as constituent of eukaryal and bacterial proteins [3]. What made selenocysteine special is that subsequent efforts established the cotranslational nature of its insertion into proteins at the position of a UGA stop codon on the respective mRNA [4, 5]. Thus, selenocysteine was designated the 21st proteinogenic amino acid [6]. Discovery of pyrrolysine, lysine with ^*ε*^N in amide linkage to (4*R*,5*R*)-4-methyl-pyrroline-5-carboxylate, occurred in a different order, a single in-frame amber codon within the gene encoding the monomethylamine (MMA) methyltransferase in *Methanosarcina barkeri* [7, 8] was later found to correspond to pyrrolysine in the crystal structure [9, 10] and have its own tRNA [11]. As pyrrolysine was also shown to be inserted cotranslationally, it was designated the 22nd proteinogenic amino acid [10]. Beside the fact that translation of selenocysteine and pyrrolysine both involves suppression of stop codons the two systems have little in common (also reviewed in [12, 13]). To emphasize the differences between the mechanisms underlying selenocysteine and pyrrolysine translation, to summarize recent insights from efforts to better understand the biology of these two unusual amino acids, and to put this knowledge into the physiological context of the unique energy metabolism of methanogenesis are the aims of this paper.

## 2. Selenocysteine and Methanogenesis

The early observation that selenium supply influences growth performance of some methanogens [14–16] is due to the fact that most of their selenocysteine-(Sec-)containing enzymes are involved in the organism's primary metabolism, methanogenesis. This process is of profound global importance as ca. 2% of the net CO_2_ fixed into biomass is recycled through methane [17, 18]. All known methanogens are members of the domain archaea. The range of substrates methanogenic archaea use for methanogenesis is rather limited reflecting the narrow ecological niche methanogens occupy; mostly simple C1- and C2-compounds such as CO_2_ (with hydrogen as reductant), carbon monoxide, methanol, methylamines (mono-, di-, and trimethylamine, as well as tetramethylammonium ion), methylsulfides, and acetate are converted to methane. The different substrate classes are metabolized via distinct, but overlapping, pathways of methanogenesis (for reviews, see [19–21]). Of the five established orders of methanogenic archaea [22], the *Methanococcales*, *Methanobacterales*, *Methanomicrobiales*, *Methanopyrales*, and *Methanosarcinales*, all (with very few exceptions) but the latter are strictly hydrogenotrophic, that is, only H_2_ + CO_2_ and/or formate serve as energy substrates.

It is intriguing that before Böck's and Stadtman's laboratories became famous for their selenium-related research they both studied (among other things) different aspects of methanogens [32–34]. It, thus, seems a striking coincidence that within the Archaea proteins containing selenocysteine [35], are until now restricted to methanogens (*Methanococcales *and the related *Methanopyrales*) [36, 37]. The known archaeal Sec-containing proteins are listed in Table 1. It should be emphasized that most methanogens do not employ selenocysteine, which poses the question as to why they get along just fine without it while others employ, and sometimes even depend on, the residue. There is no straightforward answer to this question but some considerations will be given below.

**Figure 1:**
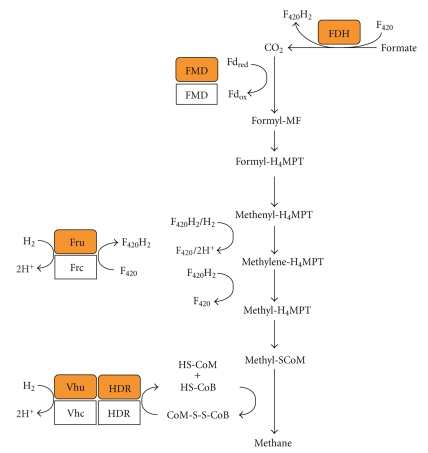
Scheme of hydrogenotrophic methanogenesis involving Sec-containing proteins (orange); the Cys-containing isoforms are in white. CoM-S-S-CoB, heterodisulfide of coenzyme M and coenzyme B; Fd, ferredoxin; Fd_ox_, oxidized Fd; Fd_red_, reduced Fd; FDH, formate dehydrogenase; FMD, formyl-methanofuran dehydrogenase; Fru, F_420_-reducing hydrogenase; F_420_, (oxidized) 8-hydroxy-5-deazariboflavin derivative; F_420_H_2_, reduced coenzyme F_420_; H_4_MPT, tetrahydromethanopterin, HDR, heterodisulfide reductase; HS-CoB, coenzyme B (*N-7-*mercaptoheptanoyl-*O*-phospho-*L*-threonine); HS-CoM, coenzyme M (2-mercaptoethanesulfonic acid); MF, methanofuran; Vhu, F_420_-nonreducing hydrogenase.

If the methanogenic growth substrate is formate, it is first oxidized to CO_2_ via (sometimes Sec-containing) formate dehydrogenase (FDH, Figure 1) [24]. In the hydrogenotrophic pathway of methanogenesis, CO_2_ is sequentially reduced to methane in seven steps via coenzyme-bound intermediates using H_2_ as the electron donor (Figure 1). The eight-electron reduction proceeds through the redox levels of formate, formaldehyde, and methanol. Formyl-MF dehydrogenase (FMD), of which a subunit (FwuB) can contain Sec [25] catalyzes CO_2_ reduction to the formyl-level and attachment to methanofuran (a 2-aminomethylfuran derivative). Of the three hydrogenases responsible for hydrogen activation in *Methanococcus, *subunits of two can contain Sec. These are the large subunit of the F_420_-dependent hydrogenase Fru (FruA, F_420_ is a hydride carrier functionally analogous to dinucleotide cofactors) [38] and two subunits of the F_420_-independent Vhu hydrogenase (VhuU and VhuD) [39]. Vhu and the heterodisulfide reductase (HDR), of which the large subunit (HdrA) can contain Sec [40], form a tight complex [41]. The heterodisulfide of coenzyme M and coenzyme B formed in the last step of methanogenesis serves as the terminal electron acceptor, which is reduced by HDR [18].

## 3. Pyrrolysine and Methanogenesis

**Figure 2:**
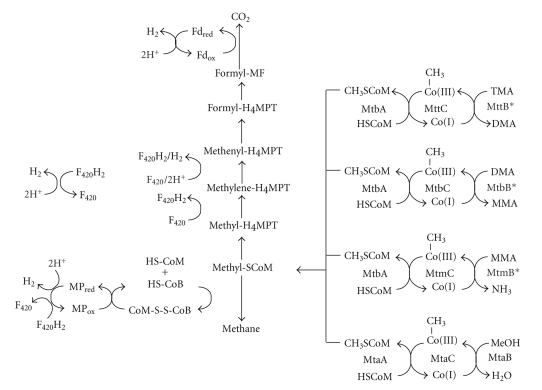
Scheme of methylotrophic methanogenesis. Methyl groups from methanol, TMA, DMA, and MMA are mobilized into metabolism by the action of a substrate-specific methyltransferase interacting with its cognate corrinoid protein in which the cofactor's cobalt ion cycles between methyl-Co(III) and Co(I) states. Methyltransferases that are pyrrolysyl-proteins are marked with an asterisk. The corrinoid cofactor is then demethylated by the action of a methylcobamide:CoM methyltransferase such as MtbA (for methylamines) or MtaA (for methanol). Adventitious oxidation can inactive the corrinoid proteins to the Co(II) state, which can be reductively reactivated by RamA (for methylamines) and possibly by RamA homologs for other pathways. Reducing equivalents in the form of hydrogen, F_420_H_2_, or Fd_red_ from the oxidation of methyl-CoM are used to reduce methanophenazine (MP) and subsequently CoM-S-S-CoB, thereby generating ATP via electron transport phosphorylation and the free HS-CoM and HS-CoB cofactors; CoM-S-S-CoB is then recycled by the reduction of methyl-CoM to methane. See Figure 1 for cofactor abbreviations.

The *Methanosarcinales* have the most diverse catabolism of all methanogens, largely due to the ability to use methylated substrates. Methylotrophic methanogenesis requires simultaneous oxidation and reduction of the methyl group (Figure 2) on the same C1 carriers employed during hydrogenotrophic methanogenesis. The reducing equivalents from the oxidation of one methyl group, at least some of which are produced as hydrogen [42], are used to reduce three methyl groups to methane. Several steps of both the reductive and oxidative branches of methylotrophic methanogenesis may rely on distinct isozymes from those essential for acetotrophic or hydrogenotrophic methanogenesis [43]. Both oxidative and reductive branches originate with methyl-Coenzyme M (methyl-CoM); thus, key to the diverse substrate range of *Methanosarcinales* are a number of methyltransferases with specificity for a particular methylated substrate such as methanol [44, 45], MMA [46], dimethylamine (DMA) [47], or trimethylamine (TMA) [48]. Each methyltransferase methylates a dedicated cognate corrinoid protein, subsequently demethylated by one of several corrinoid protein:CoM methyltransferases, such as MtbA, or MtaA [49]. An iron-sulfur protein, RamA, reductively activates the methylamine corrinoid proteins prior to initial methyl transfer [50]. The corrinoid proteins are homologous to the B_12_-binding domain of methionine synthase [7, 8, 51, 52] and share the double Rossman fold which binds the cofactor, while the corrinoid:CoM methyltransferases are closely related to uroporphorinogen decarboxylases [51, 53]. Both corrinoid proteins and CoM methylases are often erroneously classified as their more commonly known homologs in genome annotations. In contrast, the methanol, MMA, DMA, and TMA methyltransferases share no sequence similarity with each other [7, 8]. However, both the methanol [54] and MMA methyltransferases [9] have TIM barrel structures in common with methyltransferases interacting with homologous corrinoid proteins [55].

Multiple isoforms of each type of methyltransferase and cognate corrinoid protein are often encoded in different *Methanosarcinales *genomes [56–59]. Mutagenesis of all three methanol methyltransferase genes is required to eliminate methanol dependent growth in *Methanosarcina acetivorans* [60]. Although two MMA methyltransferase genes (*mtmB1* and *mtmB2*) are found in *M. barkeri *MS, only MtmB1 is isolated as an abundant protein during growth on MMA [46, 61]. The *mtmB2* gene of *Methanosarcina mazei* is most highly expressed during growth on methanol, possibly representing a nitrogen scavenging strategy [62]. MttB1, the predominant TMA methyltransferase isoform [48, 61], may preferentially interact with a TMA-specific permease in *Methanococcoides burtonii* [63].

The abundance of the isolated methylamine methyltransferases added to the initial surprise of finding a single in-frame amber codon in *mtmB1* in two different strains of *M. barkeri* [7]. An amber codon was also found in the *M. barkeri mtbB1*, *mtbB2*, and *mtbB3* genes encoding isozymes of DMA methyltransferase, and the *M. barkeri* and *Methanosarcina thermophila* TMA methyltransferase gene *mttB* [8]. The genomes of *M. barkeri*, *M. acetivorans*, *M. mazei*, and *M. burtonii* have since shown the amber codon is a conserved trait in all methylamine methyltransferase genes [56–59]. In each class of methyltransferase the amber codon placement is different, but conserved in genes encoding isozymes of a particular methyltransferase. A single exception is the *mttB3* gene of *M. burtonii* that lacks an in-frame amber codon [63]. This gene is not expressed during growth on TMA [63], but may instead be specific for a known or unknown methylotrophic substrate. For example, the encoding gene for tetramethylammonium chloride methyltransferase is not yet identified [64]. The UAG codon remains in methylamine methyltransferase transcripts [8], yet little detectable UAG termination product of *mtmB* is detectable in cell extracts [65, 66]. Peptide sequencing of HPLC isolated peptides revealed the reading frame conserved before and after the UAG-encoded position [8, 65], with a lysine observed at the UAG-encoded position. Lysine codon usage is normal in other genes in *M. barkeri*, and the possibility that a labile lysine residue could be present at the UAG position that was destroyed during peptide isolation [65] was addressed by the structure of MtmB and the subsequent visualization of pyrrolysine [9, 10]. Mass spectroscopy of MtmB, MtbB, and MttB confirmed the mass of pyrrolysine corresponding to the proposed structure at the UAG encoded position of all three proteins [61].

## 4. Peculiarities of Archaeal Selenocysteine Synthesis and Incorporation

The pathway of Sec biosynthesis and incorporation is well understood in *E. coli* [67]. First, Sec-specific tRNA (tRNA^sec^) is charged with serine by seryl-tRNA synthetase, and the seryl moiety is subsequently converted to a selenocysteyl-moiety by Sec synthase (SS). The selenium donor is selenomonophosphate generated by selenophosphate synthetase (SPS). The specialized translation elongation factor SelB (homologous to EF-Tu) delivers in its GTP-bound form the selenocysteylated tRNA to the ribosome via binding of a secondary structure on the selenoprotein mRNA, the SECIS element, located immediately adjacent to the UGA codon [68–71]. This binding triggers a conformational change in the quaternary Sec-tRNA^sec^-SelB-GTP-SECIS complex, which allows for insertion of the charged tRNA into the ribosomal A site [72, 73].

**Figure 3:**
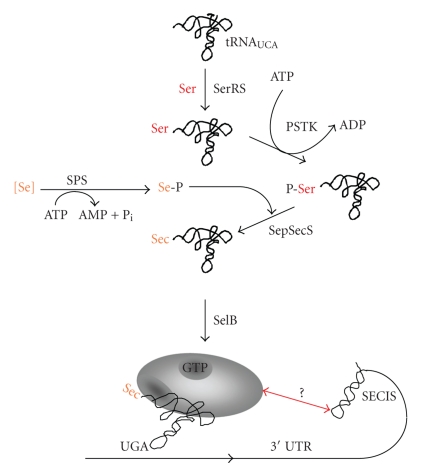
Schematic representation of selenocysteine biosynthesis and incorporation in Archaea. 3′UTR, 3′-untranslated region; PSTK, seryl-tRNA^sec^ kinase, [Se], reduced Se-species; SelB, Sec-specific elongation factor; SepSecS, *O*-phosphoseryl-tRNA^sec^:selenocysteine synthase; Ser, serine; Se-P, seleno(mono)phosphate; SerRS, seryl-tRNA synthetase; SPS, selenophosphate synthetase; see text for details.

Both Sec synthesis and Sec insertion differ in Archaea from the bacterial path (Figure 3). In fact, identical strategies appear to be employed by archaea and eukarya—to the exclusion of bacteria. Conversion of the seryl-moiety to Sec proceeds via two steps: seryl-tRNA^sec^ is phosphorylated to *O*-phosphoseryl-tRNA^sec^ in an ATP-dependent reaction by phosphoseryl-tRNA^sec^ kinase (PSTK) [74, 75]; subsequently, the *O*-phosphoseryl-moiety is converted to the Sec-moiety by* O*-phosphoseryl-tRNA^sec^:Sec synthase (SepSecS) [76]. SepSecS (also named SecS in the eukaryal system [77]) is (like SS) a pyridoxal phosphate-dependent enzyme, and its proposed reaction mechanism is analogous to that proposed for bacterial SS [76, 78]. Although it has been shown for *Trypanosoma brucei* that only the PSTK- and SepSecS-dependent pathway is present [79], and although high-resolution structures of both enzymes have recently become available [80–84], the physiological function of *O*-phosphoseryl-tRNA^sec^ and, thus, the selective advantage for investing an additional ATP in Sec synthesis (as compared to the bacterial system) is not evident. The possibility that this pathway renders activation of selenium in the SPS reaction unnecessary [85, 86], turned out not to apply [29]. Severely selenium deprived rats were shown to incorporate cysteine at the selenocysteine-position of selenium-dependent thioredoxin reductase, probably in order to salvage at least some enzymatic activity [87]. Furthermore, it was suggested that *O*-phosphoseryl-tRNA^sec^ might be converted to cysteinyl-tRNA^sec^ in *Methanococcus maripaludis* [88]. However, if no highly stringent mechanisms to ensure the fidelity of codon/amino acid correlation during translation under “normal” physiological conditions were operative, such a possibility would render any UGA codon within a selenoprotein mRNA ambiguous for the amino acid to be inserted, which in turn would have detrimental effects on the “mistranslated” protein's activity. Fortunately, *M. maripaludis*, for which this scenario was proposed, is the ideal model to study it and exciting new insights regarding the physiological meaningfulness of the additional phosphorylation step await us. The same is true for the *in vivo* role of selenium-binding protein (SeBP), a 81 amino acid polypeptide which binds one reduced selenium per tetrameric protein *in vitro* [89, 90] and could therefore be involved in transport and intracellular trafficking of selenium.

In eukarya and archaea, the SECIS element is located in the nontranslated regions of selenoprotein mRNAs; noteworthy, there is no relation between the SECIS elements in terms of structure and/or sequence, which indicates that they have distinct evolutionary origins [67] and that the modes of SECIS-function, that is, SECIS recognition, might differ. While the bacterial SECIS is specifically bound by the Sec-specific translation elongation factor SelB, the archaeal and eukaryal counterparts are not; there, the respective SelBs do not contain a C-terminal extension shown to be responsible for SECIS-binding in *E. coli* [91–93]. Instead eukaryal SECIS elements are bound by “SECIS-binding protein 2” (SBP2) and the ribosomal protein L30 [94, 95]. Other factors have also been shown to be involved in SECIS-dependent UGA recoding, but their exact function is not clear yet (current knowledge is summarized in [96, 97]). So far, no SECIS-binding factor has been identified in Archaea. Homologs of SBP2 are not encoded in any available archaeal genome and the two L30 homologs of *Methanococcus maripaludis* appear not to be involved in selenocysteine insertion because their homologous overproduction had no effect on selenoprotein formation of the organism (Sattler and Rother, unpublished data). Further, *Methanocaldococcus jannaschii* encodes three L30 homologes [40] and the one most similar to SBP2 was tested whether it could function in eukaryal selenoprotein synthesis but did not [94]. This may not be too surprising as both the structure/sequence and the distance of the archaeal SECIS elements to the respective UGA codons they recode is different to the eukaryal SECS elements [67], which may be as far as 5.4 kb downstream of their cognate UGA [98] with an average distance of 500–2,500 nt [30, 99]; the archaeal SECIS structures are usually located in closer proximity (80–1,300 nt [40, 100, 101]). It is possible that a distance constraint is the reason for a unique situation regarding the selenoprotein FdhA (encoding a subunit of formate dehydrogenase; see Table 1); the putative SECIS element is located in the 5′-nontranslated region of the respective deduced mRNA, maybe because the distance of the Sec codon and the 3′-nontranslated (>1,600 nt) region would be too great. However, this scenario needs to be verified by experimentation and fortunately, the tools required for such an *in vivo* analysis are now available [102, 103].

## 5. Pyrrolysine Synthesis and Incorporation Is Different from Sec

An amber decoding tRNA^Pyl^ (then called tRNA_CUA_) was identified as the product of the *pylT* gene [11] simultaneously with the discovery of pyrrolysine. This gene is the first of the *pylTSBCD* gene cluster found in representative *Methanosarcinales* [11]. The five *pyl* genes have proven sufficient for the biosynthesis and genetic encoding of pyrrolysine [66].

**Figure 4:**
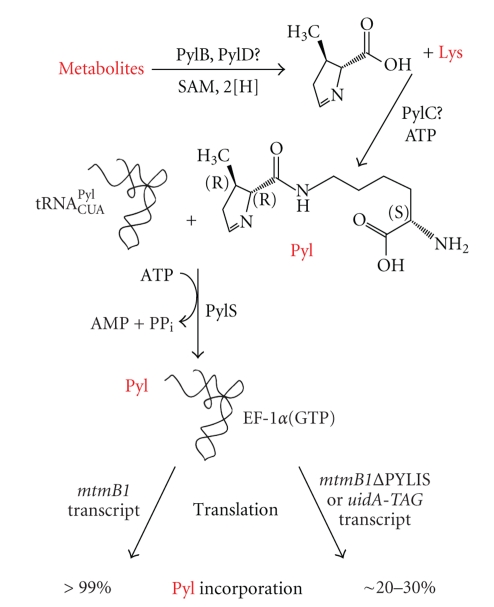
Schematic representation of pyrrolysine incorporation into protein. The *pylB*, *pylC*, and *pylD* genes have been shown to enable pyrrolysine biosynthesis in *E. coli*, but their exact roles are unknown. A conceptual and speculative scheme is shown which is keeping with the activities of proteins in their respective proteins families. Lysine is likely to form the acyl of pyrrolysine, but may also be coupled to an early precursor which subsequently cyclizes after amide bond formation. Pyrrolysine is given entrance to the genetic code by PylS in Archaea, the equivalent in Bacteria are the products of the split gene *pylSc* and *pylSn*. The direct formation of pyl-tRNA^Pyl^ is likely followed by binding to the elongation factor used by the canonical amino acids, that is, EF-1*α* in Archaea. The common bacterial elongation factor EF-Tu can bind charged tRNA^Pyl^ both in vivo and in vitro. The recognition of pyl-tRNA^Pyl^ by non-specialized elongation factors underlies the relatively high level of UAG translation in a reporter gene such as *uidA* with an introduced amber codon in organisms having tRNA^Pyl^, PylS, and pyrrolysine or an analog to charge tRNA^Pyl^. The PYLIS is not essential for this level of translation, as shown by replacement of PYLIS in *mtmB1*, but may enhance UAG translation to some extent. This effect is unlikely to require the structure formed by PYLIS. See text for further details.

Initial scenarios suggested that tRNA^Pyl^ might be charged with lysine, either by a complex of class I and class II lysyl-tRNA synthetases [104], or by the *pylS* gene product, identified as a homolog of class II aminoacyl-tRNA synthetases [11]. Both ideas proved incorrect. Loss of the genes encoding one or the other LysRS from *Methanosarcina acetivorans* does not affect UAG translation as pyrrolysine [105], and the substrate of PylS is not lysine, but pyrrolysine itself [106]. Quite unexpected by analogy to selenocysteine, pyrrolysine is not made on tRNA^Pyl^, but as a free amino acid which is directly ligated to the tRNA (Figure 4).

PylS is a pyrrolysyl-tRNA synthetase, as shown by *in vitro* activity with chemically synthesized pyrrolysine [10, 107, 108]. The 50 *μ*M K_m_ of PylS for stereochemically pure pyrrolysine remains the lowest observed for any substrate or pyrrolysine analog tested [107]. The function of the *pylS* and *pylT* gene products was confirmed by *in vivo* synthesis of pyrrolysine-containing proteins in *E. coli* transformed with *pylT* and *pylS* and supplemented with exogenous pyrrolysine [107]. As an orthologous pair, PylS and tRNA^Pyl^ have been exploited in recent years to incorporate Pyl analogs with modifiable tags for production of recombinant proteins with derivatizable residues [109–111]. Several structures of the catalytic core of *M. mazei* PylRS enzymes are now available, although these lack the first 180 residues of the protein [112–115]. The activated pyrrolysyl-adenylate made prior to ligation to tRNA^Pyl^ lies in a deep groove with the pyrroline ring buried in a hydrophobic pocket [113, 114]. A mobile loop can bring a tyrosine into H-bonding distance of the imine nitrogen of pyrrolysine [113], but is not essential for activity [114]. Analogs of pyrrolysine lacking an electronegative group at this position are recognized with a lowered specificity constant [116]. Mutation of the hydrophobic pocket has led to enhanced activity with a derivatizable pyrrolysine analog [117], or ^*ε*^N-acetyl-lysine [118].

In contrast to the single archaeal *pylS* gene, bacteria such as *Desulfitobacterium hafniense* encode *pylS* in two separate genes, with the catalytic domain encoded by *pylSc* and the N-terminal region encoded by *pylSn *[11, 13, 106]. PylSc is competent *in vitro* as a pyrrolysyl-tRNA synthetase, but has minimal detectable activity *in vivo* [115, 119]. This may be due to high affinity binding of tRNA^Pyl^ by PylSn which presumably interacts with PylSc (Jiang and Krzycki, manuscript in preparation). The structure of PylSc and tRNA^Pyl^ complex [115] revealed the compact core of tRNA^Pyl^ enhancing interaction with the catalytic domain. Interestingly, unique elements of tRNA^Pyl^ such as the elongated anticodon stem, small variable loop, and T-loop without the classical T*ψ*C sequence [11, 120], were not directly contacted by PylSc.

Recombinant expression of *pylTSBCD* in *E. coli* results in translation of UAG as internally biosynthesized pyrrolysine in reporter proteins [66]. Transformation of only *pylBCD* leads to pyrrolysine production, as determined by PylS-based charging and amino acid activation assays [66]. The recombinantly produced amino acid comigrates with synthetic pyrrolysine in TLC (Gaston and Krzycki, unpublished data). The enzymatic activities of the pyrrolysine biosynthetic genes, that is, PylB, PylC, and PylD are yet unknown but they share homologies that lead to logical possibilities [11, 66, 121]. The *pylB* gene product is highly similar to biotin synthase, while lacking key residues for binding dethiobiotin but it has all other hallmarks of the radical SAM family whose members catalyze various intramolecular rearrangements, reductions, and methylation reactions [122]. PylB may catalyze formation of the methylated ring or ring precursor. The *pylC* gene product is related to the carbamoyl-phosphate synthetase and D-alanine-D-alanine ligase superfamily and might play a role in amide bond of pyrrolysine between lysine and the ring precursor. PylD has an NAD-binding signature, and could be involved in formation of the imine bond.

The ability of a single gene cluster, *pylTSBCD,* to transform a naïve organism to incorporate genetically encoded biosynthesized pyrrolysine could underlie the far-flung distribution of pyrrolysine genes in microbes [66]. Although only *Methanosarcinales* are known to have *pyl* genes in the Archaea, all five *pyl* genes have been noted in Gram-positive bacteria such as *Desulfitobacterium hafniense* [11], *Desulfotomaculum acetoxidans*, and *Acetohalobium arabaticum* [13]. A recently sequenced genome also reveals a complete *pyl* operon in *Thermincola *sp. JR as well. Examples of *pyl* genes are also found in Gram-negative bacteria such as a *δ*-proteobacterial worm intestinal symbiont [123]. In the Gram-positive bacteria, *pylScBCDSn *typically form a single gene cluster (with the exception of *A. arabaticum* where *pylSn* precedes *pylSc*), whereas in the *δ*-proteobacterium separate *pylBCD* and *pylTScSn* gene clusters are found on different contigs of the unclosed genome sequence.

UAG appears to serve globally as both sense and stop codon in archaea having the *pyl* genes. An *E. coli uidA* gene with an introduced amber codon transformed into *M. acetivorans* resulted in mostly amber-terminated gene product, but also produced full-length active *β*-glucuronidase, complete with pyrrolysine, at an apparent efficiency of 20%–30% [124]. When this data is considered in light of the translation of *mtmB1* or reporter genes with introduced amber codons in *E. coli* dependent on *pylT* and *pylS* [66, 107, 124], it appears very likely that amber suppression underlies this relatively high level of pyrrolysine incorporation.

Sec incorporation into protein requires the presence of the SECIS element in the transcript as discussed above. An analogous pyrrolysine insertion sequence (PYLIS) element was proposed [125] whose basic structure exists in solution [126]. In the initial sequencing of the first *mtmB* and *mttB* genes, this same element was observed, but nothing similar could be seen in *mtbB* [127], a result that was further emphasized by a later more exhaustive bioinformatics study [12]. Direct replacement of the PYLIS confirmed it was not essential for incorporation of pyrrolysine into MtmB, albeit with a decrease in full-length product in the absence of the element [124]. Concomitant increase in amber-truncated *mtmB1* product indicated that some portion of the PYLIS might enhance UAG translation or diminish UAG-directed termination. The effect observed is unlikely to require the entire structure formed by the PYLIS as comparison of the PYLIS from ten methanogen *mtmB* genes shows only limited evidence of covariance [128].

Again, unlike Sec, factors that might participate in highly efficient translation of UAG as pyrrolysine during *mtmB* expression have not been identified. Two release factors are encoded in the genomes of *M. mazei* and *M. acetivorans*, which were proposed to possibly participate in UAG translation as pyrrolysine by differential recognition of stop codons [12], but only one of these homologs is found in the genomes of *M. barkeri*, *M. burtonii*, or *Methanohalophilus mahii* suggesting this could not be a general method of enhancing UAG translation. The two release factors from *M. acetivorans* were tested and one was capable of recognizing all three stop codons, whereas the other had no activity, leaving its function an open question [129].

Unlike Sec-tRNA^Sec^, Pyl-tRNA^pyl^ can be recognized by bacterial EF-Tu *in vitro* [120] and *in vivo* [107], and by eukaryotic EF-1*α*  as well [110, 111]. This would seem to obviate the need for another elongation factor, although it would be possible that an elongation factor with a higher affinity for Pyl-tRNA^Pyl^ could function in pyrrolysine-utilizing archaea or bacteria. The *pyl* genes-containing *Methanosarcinales* encode an archaeal EF1*α*, as well as a SelB homolog that could enhance recognition of Pyl-tRNA^Pyl^ [130]. This idea has not yet been tested, but it should be noted that close homologs of this same SelB-like protein could be found in other methanogens that lack *pyl* genes, indicating the role of this factor may not be connected to pyrrolysine metabolism.

## 6. Why Use Sec?

Most of the organisms for which genome information is available obviously do not employ Sec at all. Although not abided by all bioinformaticians, more than a TGA-interrupted gene and an orphan translation factor are needed to conclude that an organism synthesizes selenoproteins [131]. The simplest way would be to conduct an experiment but at least tRNA^sec^ and the Sec-biosynthesis factors (SS or PSTK/SepSecS) have to be encoded as well.

Why organisms use Sec is still not understood, mostly because for most characterized selenoproteins the specific functions of Sec are still unknown. Furthermore, for all but one of the selenoproteins of prokaryotes (clostridial glycine reductase), homologous proteins with cysteine (Cys) at the respective position exist [132]. This is true even within one organism in Sec-utilizing methanogens. In *M. maripaludis *strain JJ, for example, all of its selenoproteins are dispensable during growth with H_2_ + CO_2_ because they can be substituted by a set of Cys-isoforms. On the other hand, a very close relative, *M. maripaludis* strain S2, cannot do without its selenoproteins, probably because for at least one of them no complementing Cys-isoform exists or is sufficiently active [29]. It was shown for strain JJ that the Cys-isoforms are present at a much higher level than the selenoproteins they replace, which was interpreted as a means to counteract decreased kinetic efficiency of the Cys-containing proteins in order to retain competitive substrate flux through the methanogenic pathway [133]. Thus, using selenoproteins could be a strategy to avoid unnecessary protein synthesis. However, the notion that selenoenzymes are “super-cysteine-enzymes”, an argument often used and derived from the observed drastically lower enzymatic efficiencies of Sec to Cys mutant variants of selenoproteins [134, 135], is proven to not always be correct [136]. It can, thus, not be the sole explanation for the use of Sec. The same is true for the argument that Sec is more reactive than Cys due to the fact that the selenol group is mostly deprotonated at physiological pH while the thiol group is mostly protonated due to the different p*K*_a_ values (5.2 for Sec, 8.3 for Cys) [137, 138]. However, measuring the respective values in different Cys- and Sec-containing peptide- and protein-contexts showed that p*K*_a_ cannot serve as the sole explanation for the use of Sec [139, 140]. Selenoproteins have a lower redox potential than their Cys-homologs in the cases where this was determined [141, 142], a feature that is also often used to explain the use of Sec. It was recently pointed out that Sec has a higher nucleophilic character than Cys and as a consequence might better facilitate initial high rates in redox catalysis [143]. On the other hand, it has been argued that higher electophilicity of Sec than Cys and ultimately protection of a Sec-containing enzyme from overoxidation could be the “biological rationale” to employ Sec [144]. However, all these arguments may be true for some, but most probably not for all Sec-containing proteins. Furthermore, a much more facile accessibility of the radical oxidation state of Sec as compared to Cys has been observed and although no known selenoproteins has been shown to use a radical mechanism, the electrochemical difference is remarkable. Therefore, it may have biological implications, such as in mediation of one-electron- and two-electron-transfer processes [145]. All of the mentioned potential advantages of Sec over Cys might apply for methanogenic archaea employing Sec, as most of the selenoproteins act in the central metabolic pathway, methanogenesis, and the organisms thrive at lowered redox potential conditions [146]. As such, methanogenic archaea would be ideal to study the differences between selenoproteins and their cysteine-containing isoforms in a naturally occurring system.

Today, there is broad consensus that Sec constitutes a very ancient trait, present already in the last universal common ancestor [76, 147, 148]. A simple explanation of why some methanogens use Sec and others do not is unequal loss of the trait due to different selective pressures during evolution. Closely related *Methanococcus *species, or even strains of the same species probably represent “moments” in this process [29, 149]. The Sec-utilization trait was and is being lost in archaea—and those still synthesizing selenoproteins might just thrive under conditions, like permanent absence of oxygen and low reductant concentration, which are not selecting against this trait. On the other hand, very low selenium availability should rapidly select against this trait and with typical environmental concentrations of selenate ranging from 20 nM to less than 100 pM [150, 151] such a scenario is plausible. Furthermore, microbial and chemical reduction of selenate [152, 153] and selenite [154] to insoluble elemental selenium and gaseous hydrogen selenide can deplete bioavailable selenium even further.

## 7. Why Use Pyl?

The major physiological reason apparent for pyrrolysine remains methylamine methyltransfer. The *pyl* operon lies adjacent to a separately transcribed MMA methyltransferase operon in all *Methanosarcina *species examined to date [11]. The only essential activity lost by deletion of *pylT* and the *pyl* promoter from *M. acetivorans* is the ability to use methylamines, resulting in cells unable to grow on MMA, DMA, or TMA, yet with no apparent defect in growth or methanogenesis from methanol or acetate, save that methylamines no longer are a nitrogen source [155]. This result suggests that the only viable routes to metabolism of methylamines in this organism are the corrinoid-dependent methyltransferases made via UAG translation as pyrrolysine.

To date, every bacterial species found to have a *pyl* operon has also had homologs of methylamine methyltransferase genes with conserved amber codons. Nearby genes often encode corrinoid proteins, and occasionally bacterial RamA homologs, and corrinoid:pterin methyltransferases. These genes suggest some of these bacteria have pathways to mobilize methylamine into metabolism as methylated pterins to serve as electron donors for anaerobic respirations and as a source of cellular carbon and nitrogen. Few examples exist of bacteria that use CoM (e.g., see [156]), however, in *D. acetoxidans* a cluster of genes encoding homologs of MtmB, its cognate corrinoid protein MttC, CoM methylase MtbA, and bacterial RamA are found. It is tempting to suggest this organism could employ CoM as a methyl-donor. At times, the link between methylamine metabolism and the *pyl* operon is even more intimate than seen with the methylamine utilizing methanogens. For example, the *pyl* gene cluster of *A. arabaticum* is interspersed with a gene encoding a trimethylamine methyltransferase homolog [13], and in *D. acetoxidans* an iron-sulfur protein encoded between *pylT* and *pylS* is a member of the bacterial family of proteins that are close homologs of RamA, demonstrated to activate the methylamine:corrinoid methyltransferase reaction in methanogenic archaea [50].

Homologs of the methanogen methylamine methyltransferases whose genes lack an amber codon are found in the genomes of many bacteria and a few nonmethanogenic archaea [11, 12, 123]. Such genomes always lack a complete set of *pyl* genes, unless they also possess methylamine methyltransferase genes with amber codons. Genes presumably encoding pyrrolysine-free homologs of the TMA methyltransferase are relatively prevalent, and BLAST [157] searches will readily retrieve such homologs predominantly from various *α*-proteobacteria, as well as in *Bacteriodes spp*, and the crenarchaeote *Thermofilum pendulans.* Rarer examples of DMA methyltransferase and MMA methyltransferase gene homologs lacking the amber codon can also be found. These proteins are diverged from the methylamine methyltransferases with pyrrolysine, and it remains to be seen if these genes are actual methylamine methyltransferases. The methylamine methyltransferases without amber codons have at the site corresponding to pyrrolysine a small or bulky hydrophobic residue, suggesting that these proteins will not have similar chemistry.

This begs the question as to what function pyrrolysine could serve in the demonstrated methylamine methyltransferases. Pyrrolysine brings a unique electrophilic nature to the repertoire of genetically encoded amino acids, one that can otherwise only be introduced into proteins by cofactors or residue modification [158]. Pyrrolysine reactivity with nucleophiles [9, 10] suggests the ability to participate in corrinoid dependent methylamine methyltransferase reactions by interacting with either the methylamine substrate or product. In the case of the MMA methyltransferase, pyrrolysine lies in the bottom of an anionic cleft that corresponds to the active site in corrinoid-dependent methyltransferases that are structurally analogous to MtmB [9, 10]. The pyrroline ring rotates by 90° upon forming an adduct with a nucleophile such as ammonia or hydroxylamine, and it is postulated that such a methylamine-pyrrolysine adduct could facilitate methyltransfer to the Co(I) form of the cognate corrinoid protein [9]. The ring rotation would bring the methyl-group of the MMA-pyrrolysine adduct into roughly the same position occupied prior to corrinoid transfer of the methyl-group during function of the methyl-tetrahydrofolate:corrinoid methyltransferase domain of methionine synthase [55]. Recently, site directed mutagenesis and inhibitor studies showed that pyrrolysine is crucial for rapid transfer of the methyl-group to corrinoid cofactor or protein (Longstaff, Soares, and Krzycki, manuscript in preparation). However, while these studies will demonstrate the importance of pyrrolysine in methyltransfer, many aspects of the proposed model for pyrrolysine function remain completely untested and are a priority for the field.

Pyrrolysine has been physically observed in one protein beyond methylamine methyltransferases, Thg1 from *M. acetivorans* [159, 160]. Thg1 homologs are present in a diverse number of methanogens, and in *M. acetivorans* only, the gene has acquired an in-frame amber codon that can be translated as pyrrolysine and is not essential for activity. This result is not surprising, considering *Methanosarcina acetivorans* will incorporate pyrrolysine into a recombinant bacterial reporter protein whose gene has an introduced amber codon [124]. Other examples of the ambiguity of UAG codons in *M. acetivorans* are readily found. The *pyl* genes themselves contain an example of a UAG codon serving as stop rather than sense codon. The *pylB* gene has a TAA codon ending the open reading frame in *M. barkeri*, but a TAG codon corresponds to the same position in *M. mazei*, *M. acetivorans*, and *M. burtonii*, and when the *M. acetivorans* gene is expressed in *E. coli* with TAG changed to TAA, the protein is functional in pyrrolysine biosynthesis [66]. A family of transposase genes derived from a *Bacillus *insertion element found in *M. acetivorans *and* M. mazei* [12, 56, 58] may also prove an interesting story when the functionality of these genes is investigated. Each representative has a conserved in-frame amber codon. One of the *M. acetivorans* transposase genes with an amber codon is most similar to several from *M. mazei*, indicating possible transfer of this transposase gene between species. The same family of transposases is found in *M. burtonii*, but in spite of the presence of *pyl* genes in this organism, these highly similar genes lack the amber codon [128].

## 8. Conclusion

Selenocysteine and pyrrolysine are powerful examples of the versatility inherent in the genetic code. They further provide examples of how precedent, though valuable, is not always the best predictor in scientific investigation, and that unpredicted paths can often be found as solutions for apparently similar phenomena. Selenocysteine was first thought to be another example of a posttranslationally modified amino acid, and was later found to be a genetically encoded amino acid made and incorporated into protein in a way unlike the canonical twenty amino acids. Pyrrolysine was subsequently thought to be most likely analogous to selenocysteine, yet it is biosynthesized and ligated to tRNA in a manner much more reminiscent of the common twenty amino acids. The story of each of these residues illustrates the immense information and opportunity found in the sequenced genomes, but also provides a reminder of the obligate pairing of prediction with experimental investigation.

## Figures and Tables

**Table 1:** Sec-containing proteins of archaea.

Selenoprotein-containing enzyme	Subunit	Function	Characteristic organism	Reference
Formate dehydrogenase	FdhA	Methanogenesis	*Methanococcus vannielii*	[23, 24]
Formyl-methanofuran dehydrogenase	FwuB	Methanogenesis	*Methanopyrus kandleri*	[25]
F_420_-reducing hydrogenase	FruA	Methanogenesis	*Methanococcus voltae*	[26]
F_420_-nonreducing hydrogenase	VhuD	Methanogenesis	*Methanococcus voltae*	[26, 27]
	VhuU	Methanogenesis		
Heterodisulfide reductase	HdrA	Methanogenesis	*Methanocaldococcu* *s* ^a^ * jannaschii*	[28]
Selenophosphate synthetase	homomeric	Sec synthesis	*Methanococcus maripaludis*	[28, 29]
HesB-like protein	unknown	Unknown (iron/sulfur cluster assembly?)	*Methanococcus maripaludis*	[29, 30]

^a^ *M* *ethanococcus jannaschii *was placed in a separate genus, *Methanocaldococcus* [31].
